# Thermal Injury of the Motor Branch of Ulnar Nerve Following Electrocauterization of Ulnar Artery—A Rare Complication After Open Carpal Tunnel Release

**DOI:** 10.1002/ccr3.71120

**Published:** 2025-10-03

**Authors:** Ellada Papadogeorgou, Byron E. Chalidis

**Affiliations:** ^1^ Interbalkan Medical Center Pylaia Thessaloniki Greece

**Keywords:** carpal tunnel release, motor branch, thermal injury, ulnar artery, ulnar nerve

## Abstract

We present a rare complication of open carpal tunnel release involving severe thermal injury to the motor branch of the ulnar nerve after a misplaced incision and blind electrocauterization of the lacerated ulnar artery. Accurate and precise anatomical knowledge is very important to safely perform this procedure.

## 1 Short Report

An 80‐year‐old woman presented to our department 1 month after having open carpal tunnel release (OCTR) of her dominant right hand in another institution. She reported persistent pain and discomfort in the mid‐palmar area of the hand, an inability to perform simple everyday living activities, and only slight improvement of numbness and burning sensation at the distribution of the median nerve postoperatively. The two‐point discrimination test performed comparatively at the little and index fingers was found normal for both digits, showing normal sensation in the innervation areas of the ulnar and median nerves. Froment and Wartenberg tests were positive, exposing complete motor impairment of the hand intrinsic muscles. Allen's test was also pathological, indicating ulnar artery deficiency. The electrodiagnostic study showed severe injury of the motor branch of the ulnar nerve (MUN) and signs of carpal tunnel syndrome.

**FIGURE 1:**
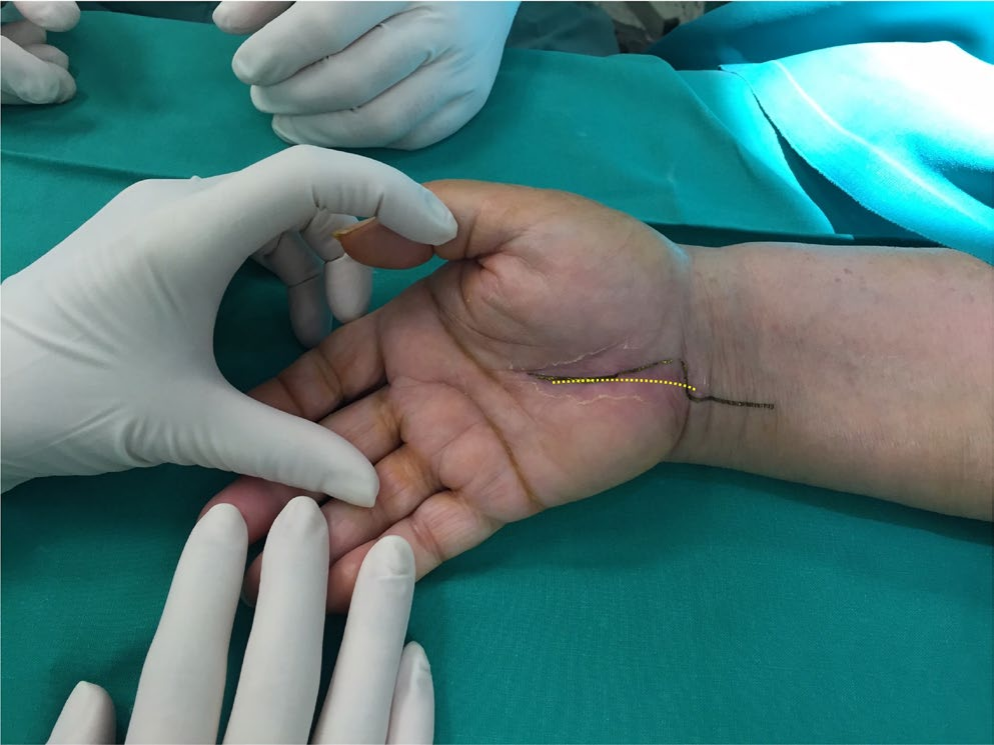
Skin incision of the extended approach (black line) following the surgical scar (yellow dotted line) from the previous complicated OCTR.

**FIGURE 2:**
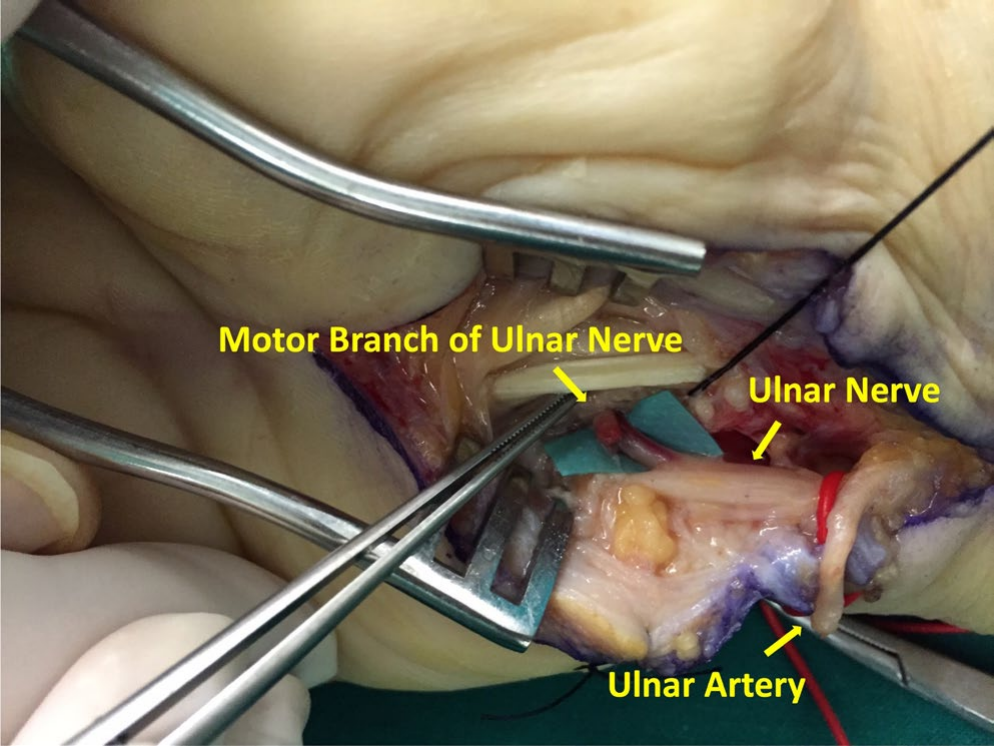
Intraoperative image of the ligated ulnar artery and thermally damaged motor branch of the ulnar nerve at the level of the ulnar nerve bifurcation, distal to Guyon's canal. The obvious discoloration of the MUN shows the extent of the injury.

Surgical exploration of the ulnar nerve was performed under general anesthesia following the previous surgical scar that was more ulnarly located compared to the typical incision of OCTR (Figure 1). The ulnar artery was found ligated, and an evident discoloration of the ulnar nerve of approximately 1 cm length was identified at the level of the hook of hamate, suggesting possible severe thermal injury of MUN by blind electrocauterization of the lacerated ulnar artery during the previous surgery (Figure 2). Intraoperative use of the nerve stimulator revealed no response from the intrinsic muscles of the hand and confirmed the damage of MUN. The Guyon canal was extensively released, and intraneural neurolysis with fascicular decompression was performed due to the advanced patient age and her refusal to undergo an additional nerve grafting procedure with an inevitable surgical donor‐site trauma. Primary MUN repair without tension was impossible. Furthermore, the transverse carpal ligament was found intact, and OCTR was completed. Wound closure was completed using 4–0 prolene suture (Ethicon Inc., Somerville, New Jersey, USA).

Postoperatively, pregabalin in a low dose of 25 mg three times per day was prescribed, and a dorsal wrist splint was applied for 4 weeks with the wrist in a neutral position. At the latest follow‐up, 9 years postoperatively, hand intrinsic muscle power was normal, and the Watenberg and Froment signs were negative. The patient was pleased with the functional outcome and was able to perform effectively all daily living activities but was troubled by frequent muscle cramps in the operated hand. Therefore, no additional procedures were required.

Although OCTR is usually a simple and safe procedure, there are various well‐described complications, with injury of the MUN being an extremely rare one. All the reported cases in the literature referred to direct unintended injury and transection of the MUN during the OCTR and not after electrocauterization [1, 2, 3]. In our case, the misplaced surgical incision, along with the surgeon's difficulty in identifying the proper anatomy, led to extensive bleeding from the ulnar artery and blind electrocauterization. If an artery is seen in the surgical field during OCTR, the surgeon should immediately check the site, as no artery normally passes through the carpal tunnel.

## Author Contributions

**Ellada Papadogeorgou:** conceptualization, investigation, methodology, writing – review and editing. **Byron E. Chalidis:** investigation, methodology, writing – original draft, writing – review and editing.

## Ethics Statement

The authors have nothing to report.

## Consent

Written informed consent was obtained from the patient to publish this report in accordance with the journal's patient consent policy and for their anonymized information to be published in this article.

## Conflicts of Interest

The authors declare no conflicts of interest.

## Data Availability

All data underlying the results are available as part of the article, and no additional data has been generated.
